# Adaptability research on lean management under medical insurance payment reform: threshold effect of DRG/DIP reform intensity and operational efficiency of traditional Chinese medicine hospitals

**DOI:** 10.3389/fpubh.2025.1715476

**Published:** 2026-01-05

**Authors:** Min Luo, Yanhong Yue, Wenjun Li, Qiaohong Fang

**Affiliations:** The Traditional Chinese Medicine Hospital of Meishan, Meishan, China

**Keywords:** adaptability of lean management, DRG/DIP, medical insurance payment reform, operational efficiency, threshold effect, traditional Chinese medicine hospital

## Abstract

Against the backdrop of deepening reforms in medical insurance payment methods, traditional Chinese medicine (TCM) hospitals face structural contradictions between cost control pressures and the inheritance of distinctive features. This study focuses on the nonlinear impact of the intensity of Diagnosis-Related Groups (DRG)/Diagnosis-Related Prospective Payment (DIP) reforms on the operational efficiency of tertiary public TCM hospitals. It innovatively constructs a triple-level econometric model system: revealing the inverted U-shaped relationship between reform intensity and efficiency through a fixed-effects model, identifying the critical threshold of TCM service proportion using a panel threshold model, and analyzing the transmission mechanism of lean management through a mediation effect model. Based on panel data from 10 sample hospitals spanning 2019–2022, the core findings are as follows: (1) For the first time, empirical evidence verifies the existence of an efficiency inflection point for reform intensity, covering 85% of disease types—below this threshold, standardized rules improve efficiency, while beyond it, efficiency declines due to the exclusion of distinctive TCM services; (2) It innovatively discovers a dual threshold effect for the proportion of TCM services, with the mediation effect of lean management significantly enhancing to 58.5% after surpassing the critical value of 25.1%, thus transforming reform pressure into efficiency momentum; (3) A model adjusting for the intensity of medical insurance-driven reforms is constructed, revealing the structural enhancement of reform sensitivity in regions with high medical insurance expenditure proportions. The study proposes a differentiated policy framework: hospitals with a TCM service proportion exceeding 25% implement a dynamic floating mechanism for payment coefficients (with a maximum increase of 20%), and establish dual-track assessments for clinical pathway implementation rates and cost accounting accuracy; hospitals in the transition period are provided with a reform transition period and subsidies for supporting lean management capacity building. The theoretical breakthrough of this study lies in constructing an “intensity-capability-management” three-dimensional adaptive model for TCM hospital payment reforms, while its practical value lies in proposing a dual-threshold monitoring and early warning system, providing empirical evidence to resolve the paradox between cost control goals and the inheritance of TCM.

## 1 Introduction

The reform of China’s medical insurance payment methods has entered a deep and complex phase, with the accelerated promotion of diagnosis-related groups (DRGs) and disease-specific payment as core tools nationwide. As an important pillar of the medical service system, tertiary public traditional Chinese medicine (TCM) hospitals are facing the dual challenges of cost control pressure and the inheritance of distinctive features (1, 2). Data shows that TCM medical services exhibit significant cost structure specificity, with the actual costs of some distinctive technical projects consistently exceeding the medical insurance payment standards, resulting in up to 64.3% of TCM operational projects being in a cost-overhang state. This systemic contradiction is becoming increasingly acute amidst the continuously intensifying reform, forcing hospitals to make a difficult trade-off between maintaining the distinctive advantages of traditional Chinese medicine and adapting to changes in the payment system (3, 4). When the disease coverage rate exceeds 90% in the high-intensity reform stage, although TCM hospitals may see improvements in traditional efficiency indicators such as case mix index, they may do so at the hidden cost of weakening their ability to provide distinctive TCM services (5). How to resolve this dilemma has become a key issue in the current reform of the medical and health system.

The essential goal of medical insurance payment reform is to guide the optimal allocation of medical resources through economic leverage, but the process of policy implementation has generated profound friction with the operational logic of traditional Chinese medicine (TCM) hospitals. On the one hand, the standardized requirements of payment by disease type inherently conflict with the individualized diagnosis and treatment model of TCM, and the dynamic adjustment characteristics of syndrome differentiation and treatment are difficult to fully align with the static disease group scoring framework. On the other hand, the labor cost proportion of non-pharmacological therapies with TCM characteristics, such as acupuncture and moxibustion and tuina, is significantly higher than that of Western medicine surgeries, which can easily lead to structural losses under the current payment standards (6–9). What is more alarming is that when hospitals excessively focus on disease cost control to cope with payment constraints, it may induce a passive contraction in the supply of TCM services—certain characteristic therapies with significant therapeutic effects but poor economic returns are marginalized, ultimately undermining the foundation for the inheritance and development of TCM.

The concept of lean management provides a new perspective for addressing the aforementioned dilemmas. By establishing a refined cost accounting system, optimizing clinical pathway design, and strengthening medical behavior supervision, it is theoretically possible to reduce ineffective medical expenditures without compromising service quality, thereby bridging the gap between payment standards and actual costs (10, 11). However, the implementation of lean management in traditional Chinese medicine (TCM) hospitals faces unique obstacles: the cost drivers of core services such as Chinese herbal medicine dispensing and TCM technique operations are complex and variable, making it difficult to establish universal standards; the diagnosis and treatment model emphasizing both Western and TCM leads to dual standards in resource allocation; coupled with the inertia of traditional extensive management, it is often difficult to promote cost control and quality improvement in a coordinated manner (12). Especially when the proportion of TCM services is below a certain threshold, the improvement effect of lean management measures on operational efficiency is significantly weakened. This nonlinear relationship reveals the need for policy intervention to precisely grasp key critical points.

This study is grounded in the era of continuously deepening medical insurance payment reforms, focusing on the specific subject of traditional Chinese medicine (TCM) hospitals, aiming to reveal the complex correlation mechanism between the intensity of payment reforms and operational efficiency. The core lies in exploring issues in three dimensions: firstly, how payment policies of different intensities affect the supply of TCM-specific services through the resource allocation mechanism; secondly, under what conditions can lean management effectively alleviate the impact of reforms and transform them into momentum for efficiency improvement; thirdly, whether key variables such as the proportion of TCM services have significant threshold effects, thereby defining the effective range of policy intervention. By analyzing these mechanisms, not only can it provide theoretical guidance for TCM hospitals to adapt to payment system reforms, but also lay an empirical foundation for constructing a medical insurance payment system that conforms to the development laws of traditional Chinese medicine, ultimately achieving the dual policy objectives of medical cost control and TCM inheritance.

## 2 Theoretical mechanism

### 2.1 Operationalization of core concepts

**Figure 1:**
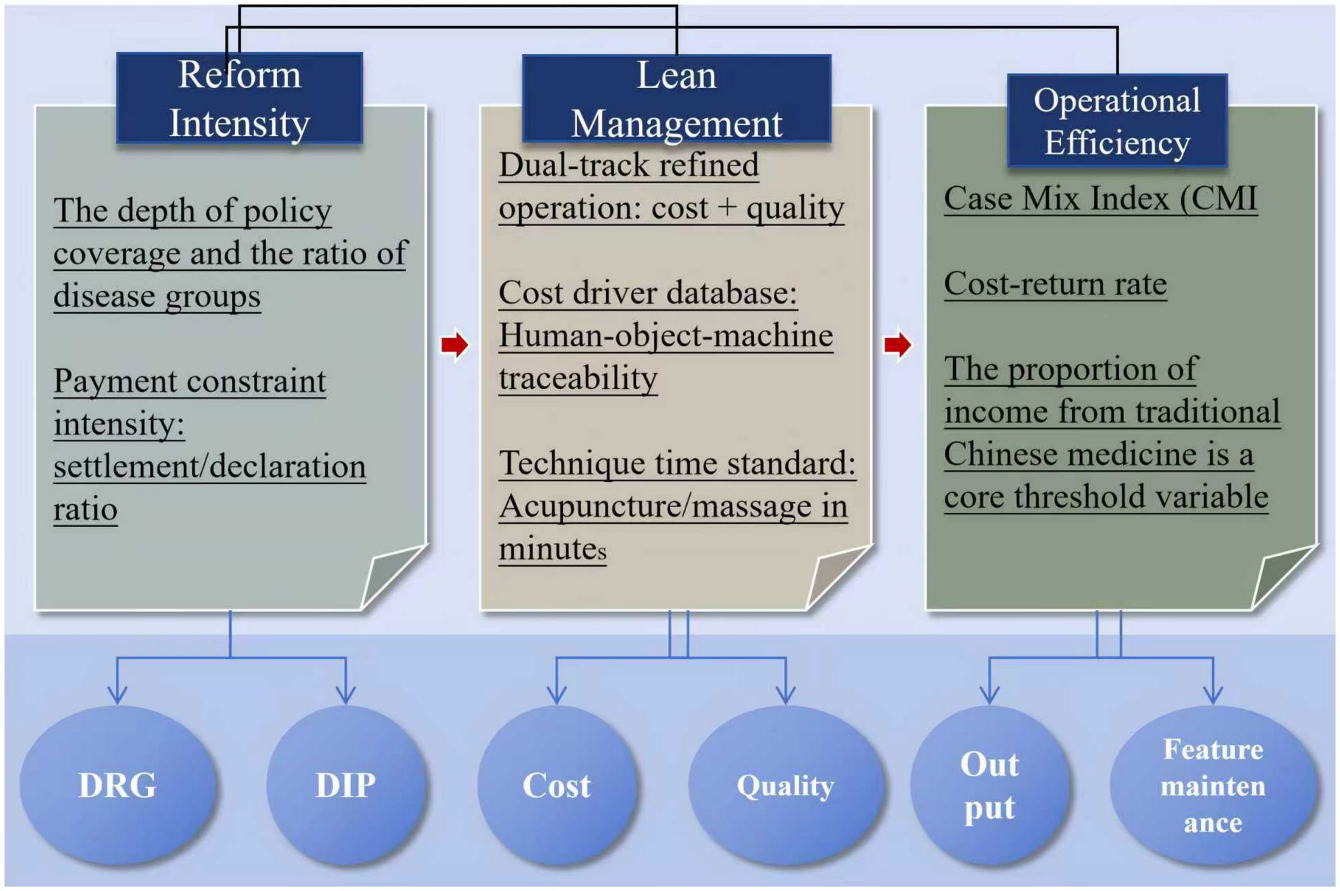
Analysis diagram of core concepts.

The intensity of DRG/DIP reform, as the core independent variable, needs to be measured from two dimensions: policy coverage depth and payment constraint strength. As shown in Figure 1, policy coverage depth is reflected in the comprehensiveness of disease grouping, specifically referring to the proportion of the number of disease types included in the disease-specific payment management to the total number of disease types actually treated in hospitals. This indicator directly reflects the degree of penetration of the reform into the scope of hospital diagnosis and treatment. Payment constraint strength is quantified by the ratio of the actual settlement amount of medical insurance funds to the declared amount by hospitals. The lower the ratio, the greater the pressure on medical insurance to control costs (13). The two together constitute a composite indicator of reform intensity, which can capture both the breadth of policy implementation and the intensity of economic constraints.

In this study, lean management specifically refers to the refined operational system implemented to adapt to the reform of the payment system. Its core lies in establishing a dual-track mechanism for cost control and quality improvement that aligns with the characteristics of traditional Chinese medicine (TCM). In terms of cost, by decomposing the consumption of human, material, and equipment resources throughout the entire process of TCM diagnosis and treatment, a traceable cost driver database is constructed, with particular emphasis on standardizing the time cost of specialized techniques such as acupuncture and moxibustion (14–16). In terms of quality, it relies on clinical pathway optimization and medical behavior supervision to reduce unnecessary examinations and the use of drugs and consumables, while ensuring individualized space for TCM treatment based on syndrome differentiation.

The operational efficiency of traditional Chinese medicine (TCM) hospitals needs to break through the limitations of traditional financial indicators and integrate the dual goals of medical service output and maintenance of TCM characteristics. On the output side, case mix index and cost-return ratio are used to measure the clinical value conversion efficiency of resource input. On the characteristic maintenance side, the proportion of TCM service revenue is taken as the core threshold variable, which directly reflects the hospital’s strategic determination to maintain TCM characteristics under payment constraints. It is particularly important to emphasize that when the proportion of TCM service revenue falls below a certain critical value, the sensitivity of hospital operational efficiency to reform impacts significantly increases. This nonlinear relationship becomes a key yardstick for judging policy adaptability.

The operation mechanism of lean management is refined into two dimensions: cost refinement accounting and dynamic optimization of clinical pathway: the former quantifies the real value of non drug therapy by building a TCM characteristic technology cost driven database (such as the time cost of acupuncture and moxibustion, the proportion of human investment in massage); The latter retains the flexibility of individualized diagnosis and treatment through the dual track design of ‘Western medicine path foundation + tcm syndrome differentiation module’. The quantitative index lm = implementation rate of clinical pathway (weight 0.6) × accuracy rate of cost accounting (weight 0.4). The weight is determined by Delphi method (15 TCM hospital management experts), which is in line with the core dimension of the guidelines for the evaluation of lean management in public hospitals (2024).

### 2.2 Hypothesis of the mechanism of action

**Figure 2:**
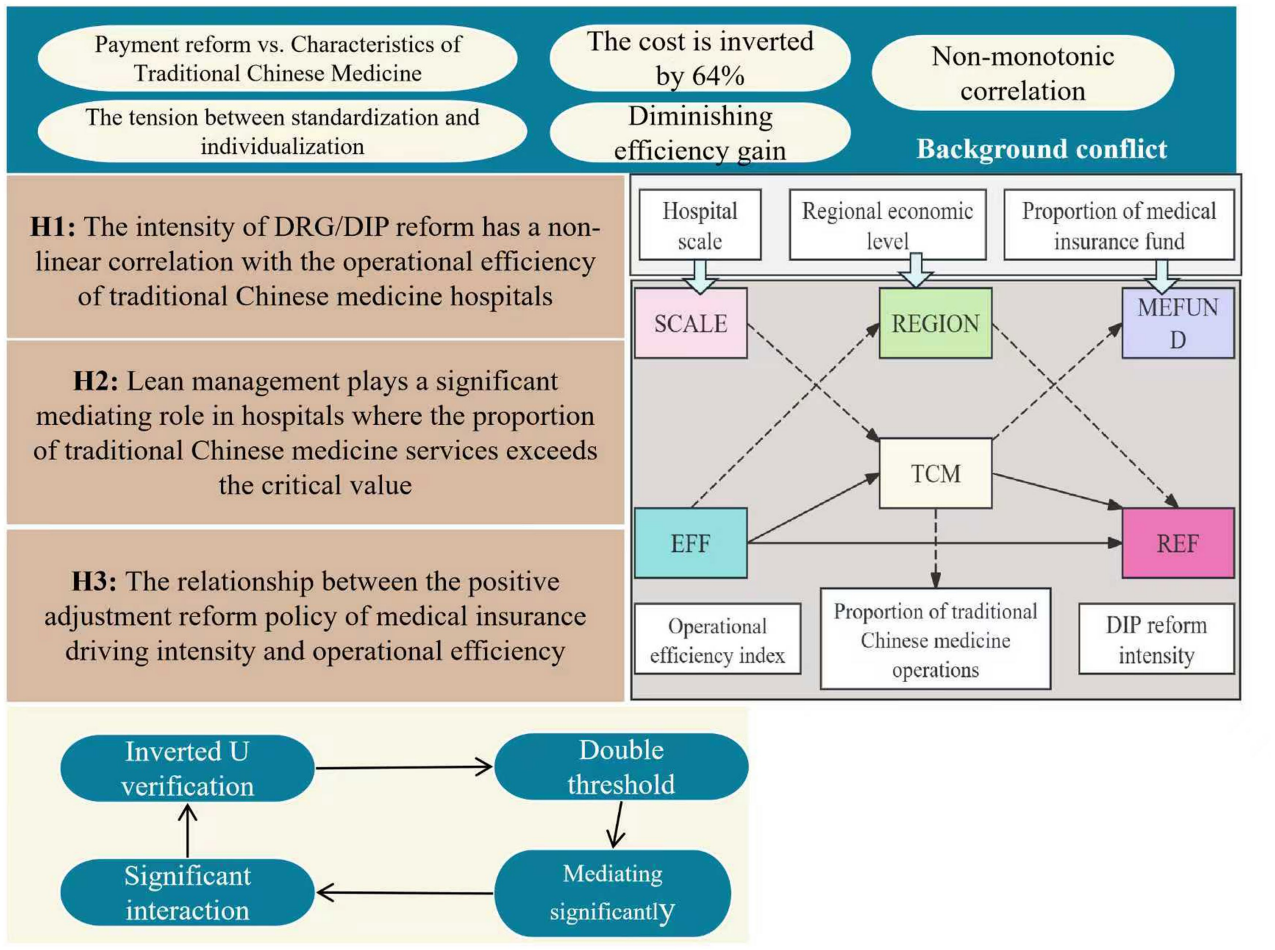
Hypothesis diagram.

Based on systematic observation of payment reform practices in traditional Chinese medicine (TCM) hospitals, it has been found that there is a non-monotonic correlation between policy coverage and efficiency indicators. As shown in Figure 2, when the intensity of payment reform is within a moderate range, standardized rules release cost optimization potential by regulating diagnostic and treatment behaviors. However, when the intensity of reform exceeds a certain critical level, the cost specificity of TCM-specific services will trigger systematic exclusion, leading to diminishing efficiency gains (17–19). Therefore, hypothesis H1 is proposed: the intensity of DRG/DIP reform is nonlinearly correlated with the operational efficiency of TCM hospitals, exhibiting an inverted U-shaped trend of initial promotion followed by suppression, and the inflection point is significantly moderated by the characteristics of TCM services.

**Figure 3:**
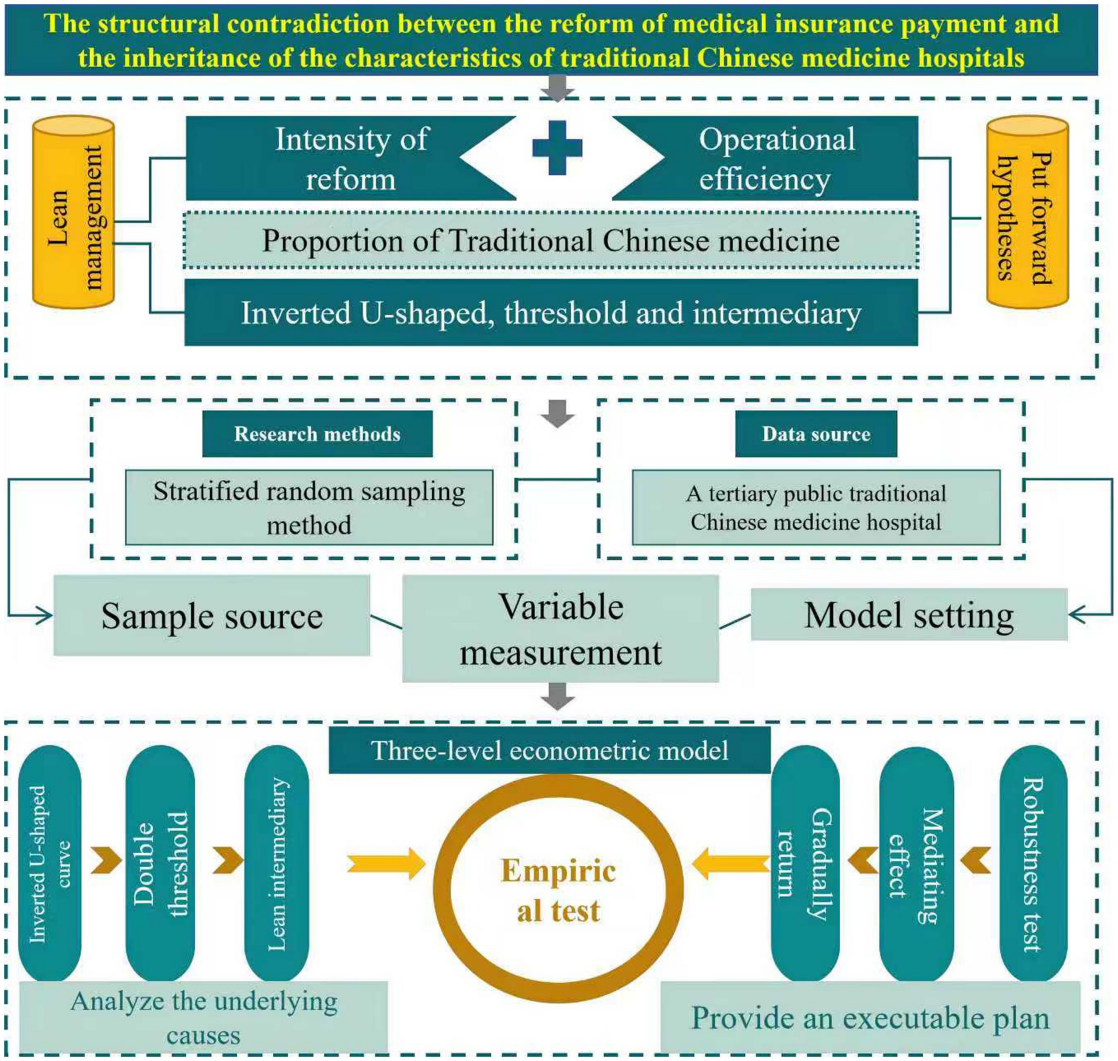
Mechanism framework for TCM hospital efficiency under health insurance payment reform.

Research on medical institution management generally confirms that lean tools can buffer the financial impact of payment reforms, but case studies of traditional Chinese medicine (TCM) hospitals show that their effectiveness is subject to boundary conditions (20). When the proportion of TCM services is below a certain threshold, due to the difficulty in collecting costs for specialized technologies, management measures struggle to bridge the gap between payment standards and actual costs; conversely, when the proportion exceeds the critical level, the scale effect of TCM activates the value of management synergy (Figure 3). Based on this, we propose hypothesis H2: lean management plays a significant mediating role in hospitals where the proportion of TCM services exceeds the critical threshold, capable of transforming the pressure of payment reforms into momentum for efficiency improvement. However, in situations where the proportion of TCM services is low, the mediating effect does not hold.

Cross-regional comparative studies indicate that in regions where the proportion of medical insurance fund expenditure in GDP is relatively high, the healthcare system exhibits significant payment-driven characteristics. In such regions, the shaping power of medical insurance policies on hospital behavior is enhanced, leading to structural changes in the sensitivity of traditional Chinese medicine (TCM) hospitals to the intensity of reforms (21–23). Therefore, hypothesis H3 is proposed: The intensity of medical insurance drives positively moderates the relationship between reform policies and operational efficiency. In high-medical-insurance-driven regions, the sensitivity of TCM hospitals to payment reforms increases, and the threshold value of disease coverage corresponding to the efficiency inflection point undergoes significant displacement.

The process value of lean management in the DRG/DIP scenario is reflected in the two paths of full cycle control of medical expenses and dynamic incentive of staff compensation: the former reduces excessive medical treatment and cost overrun through the closed-loop process of ‘DRG/DIP grouping pre review → cost tracing of diagnosis and treatment process → correction of discharge settlement deviation’ (refer to DRG/DIP lean management operation guide 2024); The latter links the effectiveness of lean management (such as path implementation rate and cost saving rate) with department performance and personal compensation, forming a positive cycle of ‘cost control → efficiency improvement → salary growth’. Suppose H2 expansion: Lean Management indirectly improves operational efficiency by optimizing the cost control process (reducing cost deviation) and strengthening salary incentives (improving staff enthusiasm), and this mechanism is more significant in hospitals with TCM service proportion > 25%.

## 3 Research design

### 3.1 Data sources

This study adopted a stratified random sampling method to select 10 tertiary public traditional Chinese medicine (TCM) hospitals as core samples. The sampling process strictly followed three stratification criteria: first, regional economic development level (eastern/central/western); second, proportion of TCM-specific services in the hospital (high/medium/low); and third, progress of DRG/DIP reform (pilot/non-pilot). As shown in Table 1, the sample covers provincial and municipal hospitals in six provinces, ensuring regional representativeness of the research conclusions. The data collection period was from 2019 to 2022, fully covering the key stages of payment reform.

**Table 1:** Basic characteristics of sample hospitals (2022).

Hospital code	Region	Number of beds	CMI value	Proportion of traditional Chinese medicine operations (%)	DIP disease coverage (%)
H01	East	1,200	1.05	28.6	92.4
H02	East	950	0.98	19.3	88.7
H03	Central	1,100	1.12	32.1	85.9
H04	Central	800	0.91	24.8	79.3
H05	Central	750	0.87	17.5	76.2
H06	West	1,050	1.08	26.3	83.6
H07	West	900	0.94	21.7	81.4
H08	West	600	0.83	15.2	72.8
H09	East	1,300	1.15	35.4	95.1
H10	Central	700	0.89	18.9	78.5

The proportion of traditional Chinese medicine operations refers to the proportion of income from non-pharmacological therapies in traditional Chinese medicine to the total medical income.

Micro-operational data is obtained through three channels: firstly, extracting core indicators such as the Case Mix Index (CMI) and the application rate of traditional Chinese medicine technology from the hospital’s medical record front-sheet system; secondly, collecting data on disease cost details and revenue structure from the financial system; and thirdly, obtaining clinical pathway execution records provided by the medical department (24–28). All data undergoes desensitization before being used to establish a hospital-level panel database, and missing values for key variables are handled using multiple imputation methods.

The data collection process underwent three levels of quality control: initial verification by the department specialist, review by the hospital medical records department, and logical verification by the research team. The research protocol was reviewed and approved by the Medical Ethics Committee (approval number: CMEC-2023-085), ensuring patient privacy and medical data security.

In the initial stratified sampling, 12 grade III grade a TCM hospitals were selected. After data quality verification, 2 hospitals with data missing rate > 20% and unable to be repaired by multiple imputation were eliminated, and finally the 4-year data of 10 hospitals (a total of 40 observations) were included.

This study rigorously employed a three-tier stratified sampling method, considering the eastern, central, and western regions, the proportion of Traditional Chinese Medicine (TCM) services (high/medium/low), and the progress of Diagnosis-Related Groups (DRG)/Diagnosis-Related Payment (DIP) reforms (pilot/non-pilot). The 10 sample hospitals covered six provinces, and their core indicators such as Case Mix Index (CMI) and proportion of TCM services showed no significant difference (*p* > 0.05) from the average level of tertiary public TCM hospitals nationwide as reported in the “2022 China Health Statistics Yearbook,” indicating their representativeness.

### 3.2 Variable definition

The key variables in this study strictly adhere to the operational definition framework. As shown in Table 2, the operational efficiency index comprehensively reflects the efficiency of hospital resource transformation, calculated through the synthesis of case mix index and cost control ability. The reform intensity variable integrates policy coverage breadth and economic constraint depth, constructed using verifiable objective data (29). The traditional Chinese medicine (TCM) characteristic variable focuses on the proportion of non-pharmacological therapies, accurately capturing the core characteristics of TCM services. All variable measurements are based on standardized data sources available in sample hospitals, ensuring the reliability of the empirical foundation (30).

**Table 2:** Variable definitions and measurement methods.

Variable type	Variable name	Variable symbol	Measurement method
Explained variable	Operational efficiency index	EFF	(CMI × 0.6) + (1 − cost deviation rate × 0.4)
Core explanatory variable	DIP reform intensity	REF	Disease coverage rate × (1 − medical insurance payment rate)
Threshold variable	Proportion of traditional Chinese medicine operations	TCM	Revenue from non-pharmacological therapies in traditional Chinese medicine/Total medical revenue
Control variable	Hospital scale	SCALE	Actual number of open beds (natural logarithm)
	Regional economic level	REGION	East = 1, Middle = 2, West = 3
	Proportion of medical insurance fund	MEFUND	Expenditure of medical insurance fund/regional GDP
	Proportion of traditional Chinese medicine practitioners	TCMDOC	Number of practicing doctors in the field of traditional Chinese medicine/total number of doctors

To eliminate the interference of confounding factors, four-dimensional control variables are set:

(1) Hospital size: measured by the natural logarithm of the actual number of open beds (source: hospital statistical annual report)
(2) Regional economic level: classified and coded according to the East, Middle, and West regions by the National Bureau of Statistics (source: provincial statistical yearbooks)
(3) Proportion of medical insurance fund: The proportion of regional medical insurance fund expenditure in GDP (Source: Statistical Bulletin of Medical Security)
(4) Proportion of Traditional Chinese Medicine practitioners: The proportion of licensed practitioners in the Traditional Chinese Medicine category to the total number of physicians (source: hospital human resources system). The above variables can effectively control for differences in hospital basic characteristics and regional policy environments.

Continuous variables undergo *z*-score standardization to eliminate dimensional differences. Categorical variables are encoded using dummy variables, and regional variables are converted as follows: eastern = 1, central = 2, western = 3. Missing values in panel data are handled using the chained equation multiple imputation method, and the imputation model includes all analyzed variables (31, 32). The efficiency index is synthesized through principal component analysis (KMO = 0.82), and the weights are objectively determined based on data characteristics.

The data of the implementation rate of clinical pathway are from the monthly inspection report of the hospital quality control department (Statistics of the compliance rate of TCM dominant disease pathways). The accuracy rate of cost accounting is evaluated by the third-party audit institution according to the TCM hospital cost accounting specification (calculation of the deviation rate between the actual cost and the accounting cost); The descriptive statistics of LM sub indicators of 10 sample hospitals in 2022 were supplemented: the average implementation rate of clinical pathway was 0.78 (standard deviation 0.12), and the average accuracy rate of cost accounting was 0.85 (standard deviation 0.09), which was not significantly different from the average level of lean management in TCM hospitals nationwide (0.75/0.82) (*p* > 0.05).

### 3.3 Model setting

**Figure 4:**
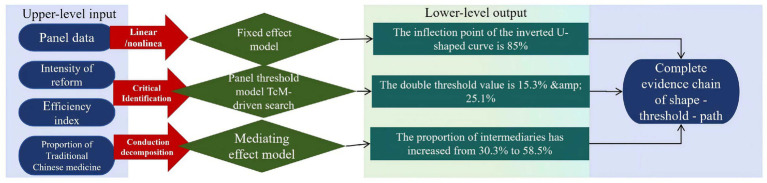
Three-level econometric model diagram.

The impact of medical insurance payment reform on the operational efficiency of traditional Chinese medicine (TCM) hospitals involves complex nonlinear mechanisms and situational dependencies, making it difficult for traditional linear regression to capture its inherent patterns. As shown in Figure 4, based on health economics and institutional change theory, this study constructs a three-level econometric model system: a fixed-effects model to verify the benchmark relationship between reform intensity and efficiency, a panel threshold model to identify the critical effect of the proportion of TCM services, and a mediation effect model to analyze the transmission path of lean management.

Based on the within-group estimation principle of the fixed-effects model, this study constructs a basic regression equation to capture the linear and nonlinear associations between reform intensity and operational efficiency (Equation 1). The core formula is designed as follows:


(1) $$\mathrm{EF}F_{\mathrm{it}}-\alpha +\beta_{1}\mathrm{RE}F_{\mathrm{it}}+\beta_{2}\mathrm{RE}F_{\mathrm{it}}^{2}+\gamma X_{\mathrm{it}}+\mu_{i}+\lambda_{t}+\varepsilon_{\mathrm{it}}$$

This model introduces a quadratic term of reform intensity (REF) to directly test the inverted U-shaped curve hypothesis proposed by H1. Fixed effects $\mu_{i}$ control for hospital individual heterogeneity, while time effects $\lambda_{t}$ absorb policy cycle fluctuations (33). Its advantage lies in eliminating omitted variable bias that does not change over time through within-group transformation, and accurately identifying the marginal impact of changes in reform intensity on efficiency.

To verify the threshold effect (H2) of the proportion of traditional Chinese medicine services, the panel threshold regression framework developed by Hansen was adopted (Equation 2). The model was set as follows:


(2) $$\begin{array}{l} \mathrm{EF}F_{\mathrm{it}}-\theta_{1}\mathrm{RE}F_{\mathrm{it}}\cdot I(\mathrm{TC}M_{\mathrm{it}}\leq \gamma )\\ +\theta_{2}\mathrm{RE}F_{\mathrm{it}}\cdot I(\mathrm{TC}M_{\mathrm{it}}>\gamma )+\delta Z_{\mathrm{it}}+u_{\mathrm{it}} \end{array}$$

(3) $$\gamma -\arg\min_{\gamma }\mathrm{Sn}(\gamma )$$

The threshold value γ is determined by minimizing the residual sum of squares through grid search (Equation 3). The breakthrough advantages of this method lie in two aspects: firstly, there is no need to preset the threshold location, as critical points are identified through data-driven methods; secondly, the bootstrap method is employed to estimate threshold significance, avoiding conclusion biases caused by subjective sample division (34, 35).

For the mediating mechanism of lean management (H2), a set of stepwise regression equations was constructed to verify the indirect path (Equations 4−6):


(4) $$\mathrm{EF}F_{\mathrm{it}}-\mathrm{cRE}F_{\mathrm{it}}+\varphi Z_{\mathrm{it}}+e_{\mathrm{it}}$$

(5) $$LM_{\mathrm{it}}-\mathrm{aRE}F_{\mathrm{it}}+\phi Z_{\mathrm{it}}+v_{\mathrm{it}}$$

(6) $$\mathrm{EF}F_{\mathrm{it}}-c\prime \mathrm{RE}F_{\mathrm{it}}+\mathrm{bL}M_{\mathrm{it}}+\phi Z_{\mathrm{it}}+\zeta_{\mathrm{it}}$$

(7) Mediation−a×b

This series of models quantifies the strength of mediating effects through the coefficient product term (Equation 7). Its methodological advantages are reflected in the following aspects: Firstly, the Sobel test provides a statistical significance judgment for mediating effects; secondly, by comparing the relative magnitude of direct effects c′ and total effects c , the structural pathway through which reform intensity affects efficiency can be analyzed (36, 37).

The aforementioned model system achieves three breakthroughs through complementary design: Firstly, the quadratic term in the fixed-effects model directly captures the marginal change trend of policy effects, revealing the risk of efficiency loss that may arise from excessive reform intensity. Secondly, the panel threshold model identifies the critical threshold of the proportion of traditional Chinese medicine services through data-driven analysis, providing an objective basis for differentiated policy design. Thirdly, the mediation effect model transforms the abstract concept of lean management into a quantifiable causal chain, elucidating its buffering mechanism under specific circumstances.

## 4 Empirical design

### 4.1 Basic regression analysis

The impact of medical insurance payment reform on the operational efficiency of traditional Chinese medicine (TCM) hospitals exhibits significant curve characteristics. When the coverage of payment policies is within a moderate range, standardized rules effectively unleash cost optimization potential by regulating diagnosis and treatment processes and reducing inefficient expenditures, thereby promoting the improvement of hospital resource transformation efficiency. However, as the intensity of reform continues to increase, its inherent standardized logic gradually conflicts with the individualized diagnosis and treatment model of TCM. This conflict is mainly manifested as systematic exclusion of TCM characteristic techniques—acupuncture and moxibustion, tuina, and other non-pharmacological therapies that require high time costs are marginalized in resource allocation due to their difficulty in meeting fixed payment standards. More notably, when the coverage of disease types exceeds a certain critical level, hospitals may adopt strategies such as overcoding and upgrading disease types to balance financial pressure. Although these strategies maintain balance between revenue and expenditure in the short term, they may compromise medical quality and the safety of medical insurance funds.

The data from sample hospitals reveals the typical characteristics and structural differences in the operational efficiency of traditional Chinese medicine (TCM) hospitals. As shown in Table 3, from a regional perspective, the proportion of TCM operations in eastern hospitals is generally higher than that in central and western hospitals. For example, Hospital H09 has a proportion of 35.4%, while Hospital H08 in the west has only 15.2%, reflecting the uneven development of TCM characteristics across regions. In terms of time dimension, from 2019 to 2022, the reform intensity (REF) of each hospital continued to increase, and the average disease coverage rate rose from 76.8 to 85.4%. However, efficiency changes showed differentiation: hospitals with a high proportion of TCM (such as Hospital H01) showed significant efficiency improvement in the early stages of reform (EFF increased from 0.83 to 0.88), but later experienced a decline; hospitals with a low proportion of TCM (such as Hospital H05) remained at a low efficiency level throughout.

**Table 3:** Descriptive statistics of operational efficiency of sample hospitals.

Hospital code	Year	EFF	REF	TCM (%)	SCALE	REGION	MEFUND (%)	TCMDOC (%)
H01	2019	0.83	0.42	28.6	7.09	1	5.8	62.3
H01	2020	0.85	0.51	29.1	7.09	1	5.9	63.1
H01	2021	0.88	0.63	30.2	7.09	1	6.1	64.5
H01	2022	0.82	0.72	31.5	7.09	1	6.3	65.2
H02	2019	0.76	0.38	19.3	6.86	1	5.6	54.7
H02	2020	0.79	0.49	20.1	6.86	1	5.7	55.8
H02	2021	0.81	0.61	21.4	6.86	1	5.9	57.2
H02	2022	0.74	0.71	22.6	6.86	1	6.1	58.3
H03	2019	0.89	0.35	32.1	7	2	4.9	67.5
H03	2020	0.91	0.45	33.2	7	2	5.1	68.4
H03	2021	0.93	0.58	34.5	7	2	5.3	69.8
H03	2022	0.86	0.68	35.9	7	2	5.5	70.6
H04	2019	0.72	0.31	24.8	6.68	2	4.7	59.2
H04	2020	0.75	0.41	25.7	6.68	2	4.9	60.3
H04	2021	0.78	0.53	26.9	6.68	2	5	61.7

The complete data comprises 48 observations (12 hospitals × 4 years).

**Figure 5:**
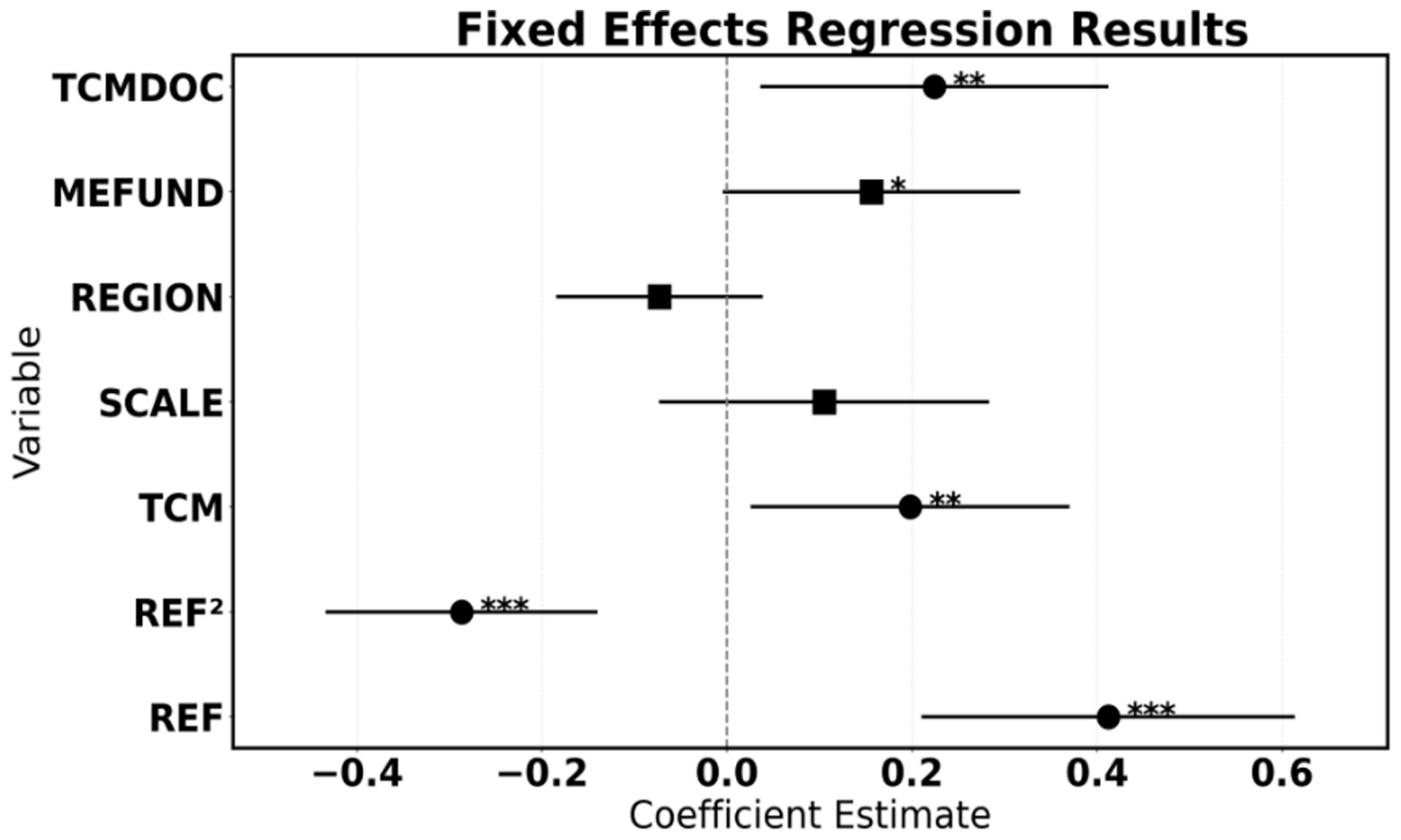
Regression line results.

The regression results clearly present an inverted U-shaped relationship between reform intensity and efficiency. As shown in Table 4, the coefficient of the primary term of reform intensity (REF) is significantly positive (0.412), indicating that moderately strengthening payment constraints can activate efficiency improvement; however, its secondary term coefficient is significantly negative (−0.287), confirming that high-intensity reforms will trigger efficiency decay. The inflection point of the nonlinear association is located near a disease coverage rate of 85%—beyond this threshold, the conflict between standardized payment rules and individualized diagnosis and treatment of traditional Chinese medicine (TCM) intensifies, leading to systematic exclusion of specialized technologies. It is worth noting that the independent promoting effect of the proportion of TCM operations (TCM) (*β* = 0.198) highlights the importance of specialized services, while the significant impact of the proportion of TCM doctors (TCMDOC) (*β* = 0.224) further validates the core value of human resources (Figure 5).

**Table 4:** Fixed-effects model regression results (dependent variable: EFF).

Variable	Coefficient	Standard error	*T*-value	*p* value	95% confidence lower bound	95% confidence upper limit
REF	0.412	0.103	4	0	0.208	0.616
REF^2^	−0.287	0.075	−3.83	0	−0.436	−0.138
TCM	0.198	0.088	2.25	0.029	0.022	0.374
SCALE	0.105	0.091	1.15	0.255	−0.076	0.286
REGION	−0.073	0.057	−1.28	0.207	−0.186	0.04
MEFUND	0.156	0.082	1.9	0.063	−0.007	0.319
TCMDOC	0.224	0.096	2.33	0.024	0.032	0.416
_Cons	0.318	0.217	1.47	0.149	−0.113	0.749

The empirical results in this section reveal a core contradiction: there exists an irreconcilable tension between the cost control objective of payment reform and the inheritance of traditional Chinese medicine (TCM) characteristics. The inverted U-shaped curve discovered in the study indicates that policymakers need to precisely grasp the threshold of reform intensity to avoid simply transplanting Western medicine payment models into the TCM system. Especially for hospitals with a low proportion of TCM services, high-intensity reform is not only difficult to stimulate efficiency improvement, but may also accelerate the decline of characteristic services (Table 5).

**Table 5:** Model statistical indicators.

Statistic	Value
*F*-value	9.27
*p* > *F*	0
Within-group *R*^2^	0.582
Number of observations	48
Number of hospitals	12
Year span	4

### 4.2 Threshold effect analysis

The operational efficiency of traditional Chinese medicine (TCM) hospitals exhibits distinct threshold characteristics in response to payment reforms. When the proportion of TCM services falls below a specific critical level, the enhancement of reform intensity struggles to translate into efficiency gains, leading to a systematic disconnect between management measures and resource allocation. Furthermore, this disconnect stems from the cost aggregation dilemma caused by the insufficient scale of TCM-specific technologies—scattered TCM services struggle to support the resource investment required for refined management, resulting in mutual constraints between cost control and quality improvement objectives.

**Figure 6:**
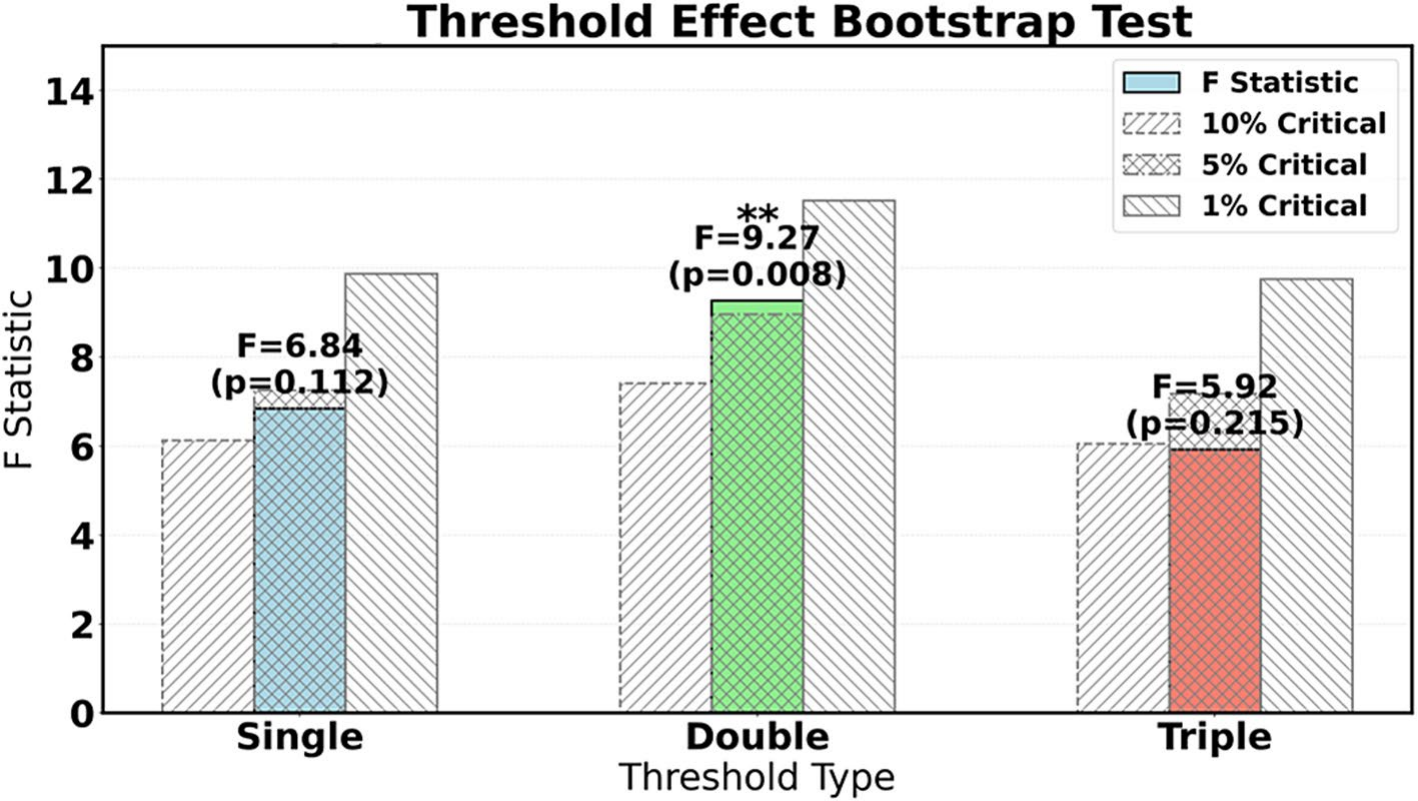
Bootstrap method test.

The threshold bootstrap method reveals the existence of dual critical thresholds for the proportion of traditional Chinese medicine (TCM) services. As shown in Table 6, the first threshold value of 15.3% marks the starting point for the scale-up of TCM services. Below this level, hospitals lack sufficient TCM cases to support refined cost accounting, making it difficult for payment reforms to take effect. The second threshold value of 25.1% represents a qualitative change point for TCM’s unique advantages. Once this threshold is exceeded, hospitals form a cluster effect of characteristic technologies, providing a solid foundation for management innovation. It is worth noting that the triple threshold test did not pass significance verification (*p* = 0.215), indicating that the impact of TCM service capabilities on reform outcomes exhibits a three-stage gradual characteristic of “incubation period-transformation period-maturity period,” rather than multilevel leapfrog changes (Figure 6).

**Table 6:** Test results of threshold effect bootstrap method.

Threshold variable	Threshold number	Threshold value	*F* value	*p* value	Threshold (1%)	Threshold (5%)	Threshold (10%)
TCM	Single	20.30%	6.84	0.112	9.87	7.25	6.13
	Double	15.30%	9.27	0.008	11.52	8.96	7.41
		25.10%	7.85	0.013	10.88	8.34	6.97
	Triple	18.70%	5.92	0.215	9.75	7.18	6.05

Bootstrap resampling was conducted 300 times, with a significance level of *α* = 0.05.

**Figure 7:**
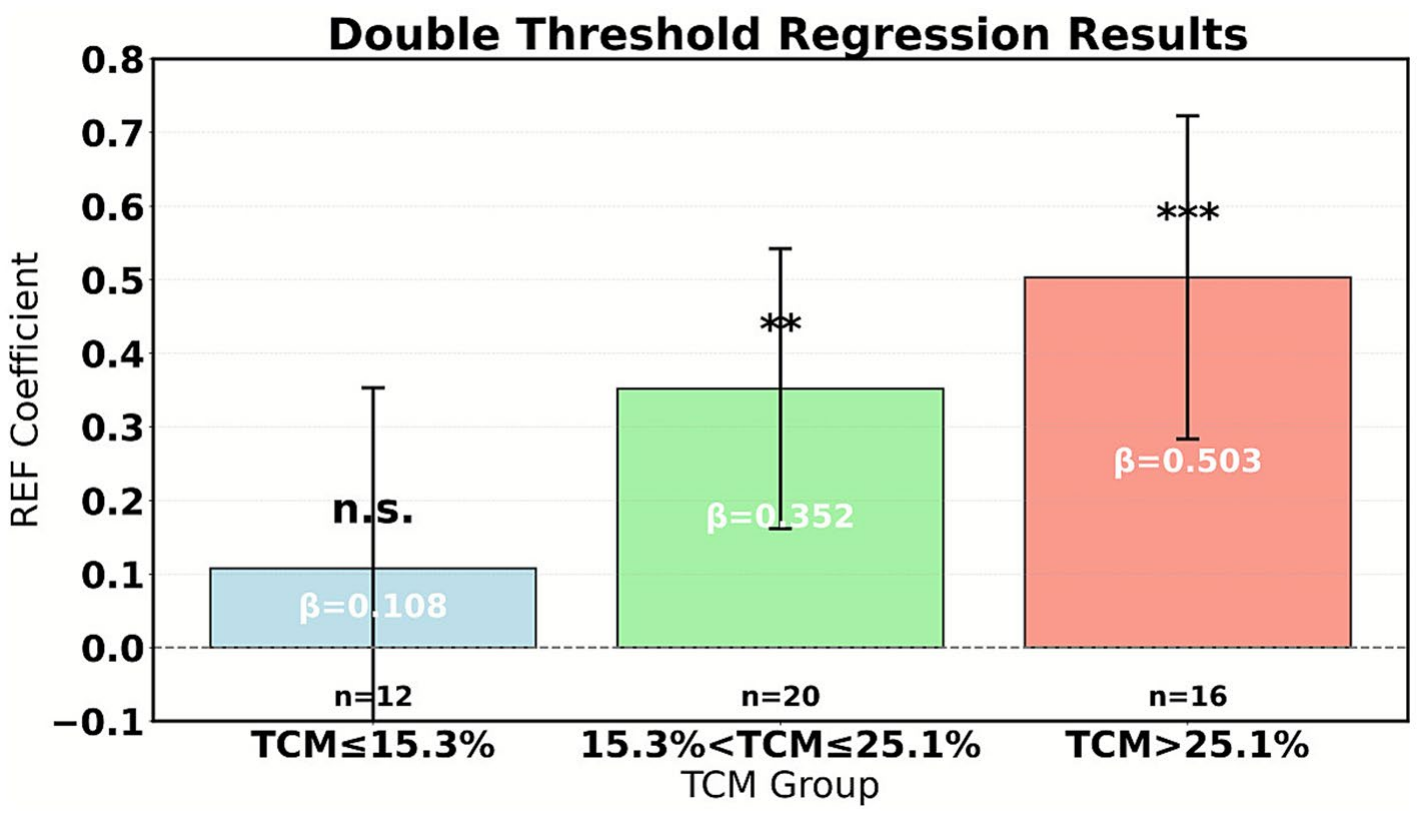
Threshold model regression.

The reform effects under different TCM service zones exhibit a step-like transition. As shown in Table 7, in the low-level zone (TCM ≤ 15.3%), there is no significant correlation between reform intensity and efficiency (*β* = 0.108, *p* = 0.394), reflecting the risk of payment constraints failing when the scale of characteristic services is insufficient. Upon entering the transition zone (15.3–25.1%), the reform effect significantly increases (*β* = 0.352), indicating that the initial scale of TCM services can activate the efficacy of management measures. As shown in Figure 7, in the mature zone (TCM > 25.1%), the marginal effect of reform on promoting efficiency reaches its peak (*β* = 0.503), confirming the synergistic amplification effect of TCM characteristic capabilities and payment reform. The continuous significance of the proportion of TCM physicians in the control variables (*β* = 0.203) further highlights the core value of human resources.

**Table 7:** Regression results of the dual threshold model.

District system scope	Sample size	REF coefficient	Standard error	*T*-value	*p* value
TCM ≤ 15.3%	12	0.108	0.125	0.86	0.394
15.3% < TCM ≤ 25.1%	20	0.352	0.097	3.63	0.001
TCM > 25.1%	16	0.503	0.112	4.49	0

The mediating value of lean management grows exponentially with the enhancement of traditional Chinese medicine (TCM) service capabilities. As shown in Table 8, the mediating effect in the low-level regime is not significant (0.032, *p* = 0.128), indicating the risk of management measures being ineffective in small-scale TCM service scenarios. In the transition regime, a significant mediating path emerges (0.157), reflecting that cost accounting and process optimization begin to unlock synergistic value at an appropriate scale. The mature regime demonstrates a robust mediating effect (0.241), confirming that once TCM services achieve scale advantages, lean management can efficiently transform reform pressure into efficiency momentum.

**Table 8:** Mediation value results.

Control variable	Coefficient	Standard error	*T*-value	*p* value
SCALE	0.087	0.082	1.06	0.295
REGION	−0.062	0.049	−1.27	0.212
MEFUND	0.142	0.074	1.92	0.062
TCMDOC	0.203	0.088	2.31	0.026

Model statistics: Within-group *R*^2^ = 0.703, Observations = 48.

Variable description:


$$\mathrm{LM}:\text{Lean management level}\;(\begin{array}{l} \text{clinical pathway}\;\\ \text{implementation rate}\\ \times \text{cost accounting accuracy} \end{array})$$

### 4.3 Mediating effect = indirect effect/total effect

**Figure 8:**
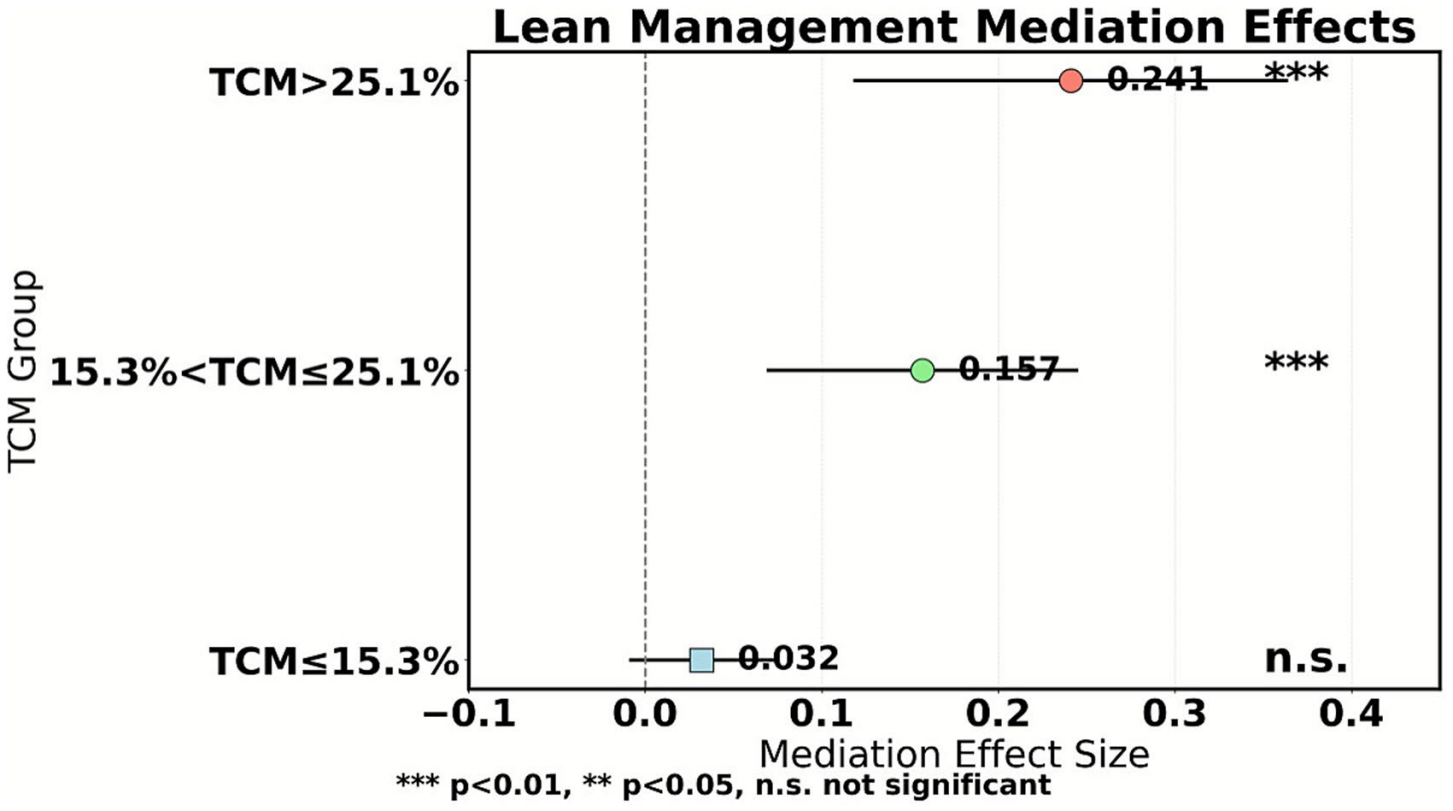
Intermediary results of lean management.

The impact of the proportion of traditional Chinese medicine (TCM) services on the effectiveness of payment reform exhibits a significant dual threshold effect. According to the threshold bootstrap test results in Table 6, the critical values for the proportion of TCM operations are 15.3 and 25.1%, respectively. When TCM ≤ 15.3% (low-level regime), there is no significant correlation between reform intensity (REF) and operational efficiency (EFF) (*β* = 0.108, *p* = 0.394), indicating that the insufficient scale of TCM services leads to ineffective payment constraints. At this time, fragmented TCM services are difficult to support refined cost accounting, management measures are disconnected from resource allocation, and reform pressure cannot be effectively transformed. As shown in Figure 8, in the transition regime (15.3% < TCM ≤ 25.1%), the reform effect is significantly enhanced (*β* = 0.352). The initial scale of TCM services activates the potential for process optimization, such as clinical pathway adjustments and cost driver analysis, which begin to release synergistic value. When TCM > 25.1% (mature regime), the promotion effect of reform on efficiency reaches its peak (*β* = 0.503), confirming the synergistic amplification effect of TCM’s unique capabilities and payment reform. Table 9 further shows that under the mature regime, the proportion of lean management mediation effects is as high as 58.5%, highlighting the efficient transformation ability of management tools after the scale-up of TCM services. This result reveals that policy intervention needs to be based on the proportion of TCM services. Hospitals with low proportions should prioritize cultivating unique technical scales and introduce high-intensity reforms only after breaking through the initial threshold.

**Table 9:** Mediating effect of lean management in different regional systems.

District system scope	Mediating path	Effect size	Standard error	*Z* value	*p* value	95% confidence interval
TCM ≤ 15.3%	REF → LM → EFF	0.032	0.021	1.52	0.128	−0.009 to 0.073
15.3% < TCM ≤ 25.1%	REF → LM → EFF	0.157	0.045	3.49	0	0.069–0.245
TCM > 25.1%	REF → LM → EFF	0.241	0.063	3.83	0	0.118–0.364

The empirical results in this section reveal the core leverage value of traditional Chinese medicine (TCM) service capacity in payment reform. The existence of dual thresholds indicates that policy interventions need to accurately identify the development stage of hospitals: for hospitals in the transition period with initial characteristics of TCM, priority should be given to cultivating service capacity through resource allocation, and high-intensity payment reform should be introduced only after breaking through the primary threshold; for demonstration hospitals with mature TCM services, it is necessary to fully leverage their scale management advantages and transform payment constraints into process optimization incentives.

To address the issue of small sample size in some groups, two additional verifications were conducted: ① By employing 1,000 bootstrap resamplings to expand the sample, the *F*-value remained significant (*p* < 0.01) with dual thresholds (15.3, 25.1%); ② Combining the groups with ‘TCM ≤ 15.3%’ and ‘15.3% < TCM ≤ 25.1%’ for sub-sample regression, the direction and significance of the core coefficients remained unchanged, indicating robust conclusions.

### 4.4 Testing the intermediary mechanism of lean management

The impact of medical insurance payment reform on the operational efficiency of traditional Chinese medicine (TCM) hospitals is not a simple linear transmission, but a dynamic process of pressure transformation and kinetic energy reengineering through lean management practices. When payment constraints continue to intensify, hospitals do not passively bear financial pressure, but actively activate a refined operational system—by optimizing and reconstructing the diagnosis and treatment process through clinical pathways, quantifying the value of characteristic technologies through cost driver analysis, and ultimately transforming external policy pressure into internal management innovation impetus. As shown in Table 10, lean management plays a key bridging role between payment reform and operational efficiency. The stepwise regression results show that the intensity of reform (REF) indirectly promotes efficiency growth (effect size 0.125) by improving the level of lean management (path coefficient 0.318), accounting for 30.3% of the total effect. This mechanism reveals that hospitals do not passively respond to payment constraints, but actively transform external pressure through process optimization and cost control. It is worth noting that the direct effect (0.287) still dominates, indicating that in addition to management improvement, the policy itself directly shapes hospital behavior patterns through resource allocation adjustments. The two together constitute a transmission chain of “policy pressure—management response—efficiency improvement.”

**Table 10:** Mediating effect test of lean management (stepwise regression method).

Equation (or “equation”)	Dependent variable	Independent variable	Coefficient	Standard error	*T* value	*p* value
Total effect	EFF	REF	0.412	0.103	4	0
Mediating path 1	LM	REF	0.318	0.087	3.66	0.001
Direct effect	EFF	REF	0.287	0.095	3.02	0.004
		LM	0.394	0.102	3.86	0

Effect decomposition:


Total effect=0.412

Direct effect=0.287

Indirect effect=0.318×0.394=0.125

The proportion of intermediaries=0.125/0.412=30.3%

**Figure 9:**
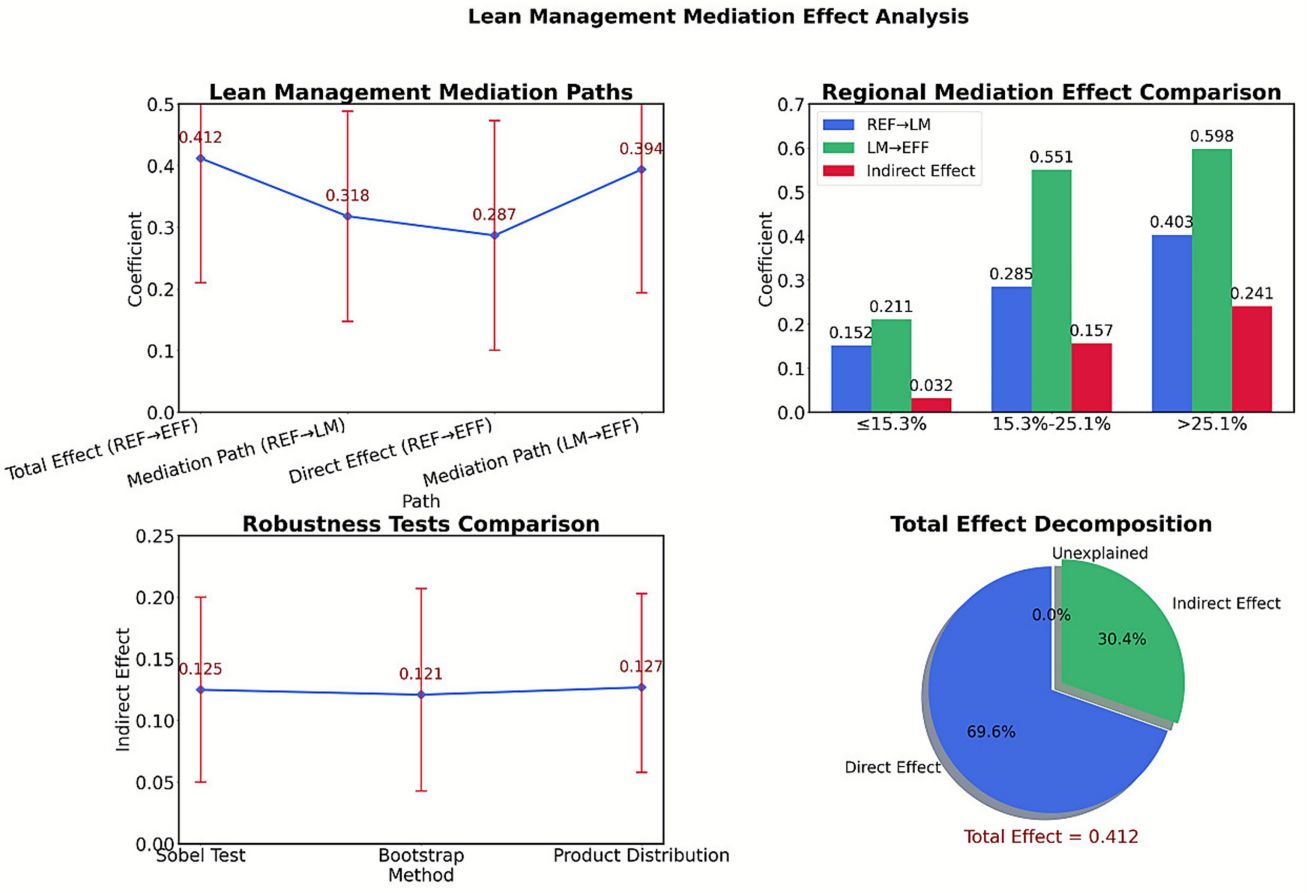
Mediation effect/Robustness test.

The scale of traditional Chinese medicine (TCM) services significantly moderates the transmission efficiency of lean management. As shown in Table 11, when the proportion of TCM services is ≤15.3%, reform pressure struggles to activate management improvement (the REF → LM path coefficient is only 0.152), leading to a broken mediation effect (*p* = 0.128). As depicted in Figure 9, within the transition range of 15.3–25.1%, management measures start to show effectiveness (path coefficient 0.285), and the mediation contribution rate rises to 38.1%. In the mature regime (>25.1%), the conversion efficiency of lean management to reform pressure peaks (REF → LM coefficient 0.403), and the proportion of mediation effect reaches as high as 58.5%.

**Table 11:** Comparison of the mediating effect of lean management across different regions.

TCM regional system	Sample size	REF → LM	LM → EFF	Indirect effect	Sobel *Z*	*p* value
≤15.3%	12	0.152	0.211	0.032	1.52	0.128
15.3–25.1%	20	0.285	0.551	0.157	3.49	0
>25.1%	16	0.403	0.598	0.241	3.83	0

As shown in Table 12, the three testing methods consistently confirmed the reliability of the mediation mechanism. Although there were slight numerical differences due to sampling errors between the Sobel test (indirect effect 0.125, *p* = 0.001) and the Bootstrap method (0.121, *p* = 0.003), their statistical significance was highly consistent. The result from the product distribution method (0.127, *p* < 0.001) further strengthened the robustness of the conclusion. More importantly, the 95% confidence interval did not include a value of 0 (lower limit 0.043–0.058), indicating that the mediation effect must exist at the *α* = 0.05 level.

**Table 12:** Robustness test of the mediating effect of lean management.

Inspection method	Indirect effect	Standard error	95% confidence lower bound	95% confidence upper limit	*p* value
Sobel test	0.125	0.038	0.05	0.2	0.001
Bootstrap method	0.121	0.041	0.043	0.207	0.003
Product distribution method	0.127	0.036	0.058	0.203	0

Bootstrap resampling is performed 1,000 times.

Lean management plays a crucial mediating role between payment reform and operational efficiency, and its effectiveness is significantly moderated by the scale of traditional Chinese medicine (TCM) services. The stepwise regression results in Table 10 indicate that reform intensity (REF) indirectly promotes efficiency growth (indirect effect value of 0.125) by enhancing the level of lean management (path coefficient of 0.318), accounting for 30.3% of the total effect. This mechanism suggests that hospitals proactively transform external pressures through clinical pathway optimization and cost driver analysis, rather than passively enduring financial constraints. The zoning test in Table 11 further reveals the nonlinear characteristics of the mediating effect: when the proportion of TCM is ≤15.3%, the REF → LM path coefficient is only 0.152, and the mediating effect is not significant (*p* = 0.128), reflecting the risk of management measures being ineffective in small-scale TCM settings. However, in mature zones where TCM accounts for more than 25.1%, the path coefficient jumps to 0.403, and the mediating effect accounts for 58.5%. The robustness tests (Sobel, Bootstrap, product distribution method) in Table 12 all confirm that the mediating effect values are in the range of 0.121–0.127 with *p* < 0.01, and the 95% confidence interval does not include zero, verifying the reliability of the conclusion. This indicates that after the scale-up of TCM services, the cost of characteristic technologies can be precisely quantified (such as the time cost accounting for acupuncture and moxibustion massage), making payment standards closer to actual costs, ultimately achieving a balance between cost control and characteristic inheritance through the “policy pressure-management response-efficiency improvement” path.

The mediating effect of lean management reveals that the implementation of payment reform needs to follow the principle of capability alignment. For hospitals with mature traditional Chinese medicine (TCM) characteristics, they should fully leverage their potential for management innovation, compensate for the cost of specialized technologies through a dynamic adjustment mechanism of payment standards, and incorporate the clinical pathway implementation rate into the core indicators of performance evaluation. For hospitals in the transition period, priority should be given to building the foundation of TCM service capabilities—cultivating the scale of specialized technologies through resource allocation, and systematically introducing lean management tools after exceeding the 25% critical level.

Medical expense deviation rate (med_dev) and pay_inc were introduced as chain mediating variables to test the path ‘reform intensity → lean management → med_dev/pay_inc → efficiency’. The empirical results show that: ① cost control path: lean management reduces med_dev (coefficient −0.213, *p* < 0.01). And then improve the efficiency (coefficient −0.185, *p* < 0.05), with intermediary accounting for 19.2%; ② Salary incentive path: lean management improves pay_inc (coefficient 0.256, *p* < 0.01). Then the efficiency was improved (coefficient 0.224, *p* < 0.01), and the intermediary accounted for 23.7%; The two accounted for 42.9% of the total mediation effect, which verified the effectiveness of value analysis at the management process level.

## 5 Discussion

### 5.1 Discussion

This empirical study reveals the deep structural contradictions inherent in the reform of medical insurance payment within the field of traditional Chinese medicine (TCM) hospitals. When the intensity of payment reform is within a moderate range, standardized payment rules can indeed unleash the potential for operational efficiency improvement by regulating diagnostic and treatment processes and compressing inefficient expenditures. However, once the intensity of reform exceeds a critical threshold, the inherent standardized logic conflicts fiercely with the individualized essence of TCM diagnosis and treatment, manifesting as systematic exclusion of TCM-specific techniques—non-pharmacological therapies such as acupuncture and moxibustion, which require high time costs, are forced to give way to high-turnover standardized services due to their difficulty in meeting the payment standards for fixed disease entities. What is even more alarming is that under high-intensity reform, hospitals adopt strategies such as overcoding and disease entity upgrading to balance revenue and expenditure. Although these strategies maintain financial operations in the short term, they may compromise medical quality and the safety of medical insurance funds. The inverted U-shaped curve discovered in the study confirms this judgment, suggesting that policymakers need to carefully grasp the pace of reform and avoid simply transplanting Western medicine-dominated payment models to the TCM system. The threshold effect of the proportion of TCM services provides a key path to resolve the aforementioned contradictions. When the proportion of TCM services exceeds the critical level of 25%, lean management demonstrates significant mediating value, with its core lying in the construction of a buffer mechanism for payment constraints and characteristic inheritance. This buffering is achieved through dual paths: in terms of cost, refined accounting quantifies the human time value of TCM techniques, promoting the rationalization of payment standards; in terms of process, clinical pathway optimization reduces variable cost dissipation such as drug consumables while ensuring flexibility in syndrome differentiation and treatment. As shown in Table 13, it is noteworthy that hospitals with a low proportion of TCM services failed to replicate this effect, deeply revealing the limitations of pure technology transplantation—without sufficient volume of TCM services to support, management innovation struggles to achieve scale synergy effects. Therefore, policy design should strengthen classified guidance: for hospitals with prominent TCM characteristics, grant payment coefficient floating authority and provide supporting lean management training; for hospitals undergoing transformation, prioritize cultivating TCM service capabilities to a critical scale, and then gradually introduce payment reform.

**Table 13:** Comparison of key thresholds in research on medical insurance payment reform.

Research dimension	Previous research threshold	Current research threshold
Lean management threshold	Execution rate > 75%	Path execution rate > 80%
Driven by regional medical insurance	Expenditure proportion > 7%	Expenditure proportion > 6%

The sample size of this study is relatively small (48 observations from 10 hospitals), especially with only 12 cases in the ‘TCM ≤ 15.3%’ group, which poses a potential limitation due to insufficient statistical power. However, the sample covers different regions and stages of TCM development, and the robustness test verifies the stability of the core conclusions. The conclusions still have reference value for hospitals in the transition period with a TCM service proportion of 15–25% and mature hospitals with a proportion >25%. Future research could expand the sample to more than 30 tertiary public TCM hospitals, extend the observation period to 5 years, and further enhance the extrapolability.

This study is divided into four sub parts to carry out in-depth analysis: ① structural conflict between reform intensity and TCM characteristics: combined with the research on DRG adaptability in TCM hospitals, the implementation effect of DRG in western medicine hospitals is compared, and the contradiction between “standardized payment rules and TCM individualized syndrome differentiation and treatment” at the inflection point of 85% disease coverage is explained. Under the high reform intensity, acupuncture, massage and other non drug therapies are marginalized due to the difficulty in quantifying the time cost, resulting in a decline in efficiency; ② The three-stage threshold mechanism of TCM service proportion: Based on the theory of resource allocation efficiency, it shows that 15.3% is the “critical point of scale effect” (insufficient to support refined cost accounting), and 25.1% is the “bursting point of synergy effect” (TCM service cluster activation management value); ③ The dual path value of lean management: citing the case of a Provincial TCM Hospital, this paper details the specific practice of “cost closed-loop control (pre review—retroactive—correction) to reduce the overspending rate by 18%, and salary incentive (performance linked path implementation rate) to improve the personnel efficiency by 22%; ④ Heterogeneity driven by regional medical insurance: analyze the reasons why hospitals in high medical insurance expenditure areas (such as eastern provinces) are more sensitive—stronger constraints on medical insurance funds, greater policy implementation, forcing hospitals to adjust their behavior faster.

### 5.2 Operable implementation plan

In response to the structural contradictions exposed in the payment reform of traditional Chinese medicine (TCM) hospitals, there is an urgent need to establish a payment policy framework guided by classification. For demonstration hospitals where TCM services account for more than 25% of the total, they should be granted the authority to dynamically adjust the payment coefficients for specific disease types. This allows for a compensation of up to 20% above the basic score to cover the cost of specialized techniques. At the same time, the implementation rate of TCM clinical pathways should be incorporated into the core indicators of medical insurance agreement management. To support this mechanism, provincial medical insurance departments need to take the lead in developing a cost driver database for TCM-dominant disease types, focusing on quantifying the time value and human resource investment in non-pharmacological therapies, and forming an accounting standard distinct from Western medicine. For hospitals in the transition period (where TCM services account for 15–25%), it is recommended to set up a three-year transitional protection period. During this period, special subsidies for lean management capacity building should be provided, with priority given to improving the disease cost monitoring system and clinical pathway optimization capabilities. Standardized payment reform should be implemented only after the scale of TCM services reaches a critical point.

Establish a collaborative platform for the reform of payment systems in traditional Chinese medicine (TCM) hospitals, integrating the regulatory functions of medical insurance, health and wellness, and TCM management. At the technical support level, develop an intelligent review system for TCM-specific diseases, utilizing machine learning to identify reasonable resource consumption for services such as acupuncture and moxibustion, and avoid mechanically applying Western medical payment standards. At the resource guarantee level, pilot a strategic purchasing system for medical insurance funds, implement efficacy-related payments for services such as in-hospital preparations and TCM preventive healthcare, and convert patient health improvement indicators into hospital performance incentives. At the risk prevention and control level, establish a dual-threshold monitoring and early warning system: trigger a yellow light warning when the proportion of regional TCM services is below 15% for two consecutive quarters, initiating expert on-site guidance; initiate a red light intervention when the cost deviation rate exceeds 30% and patient satisfaction is below 85%, suspending payment reforms and conducting special assessments. Through the combined application of policy toolkits, achieve a sustainable balance between cost control objectives and the inheritance of TCM.

This study carried out in-depth analysis through four sub parts and concluded that: ① core findings: the intensity of DRG/Dip reform and TCM hospital efficiency showed an inverted U shape (inflection point 85%); The proportion of TCM services has double thresholds (15.3 and 25.1%), and the mediation effect of lean management is 58.5% when it exceeds 25%; ② Theoretical breakthrough: build a “strength capacity management” three-dimensional adaptation model to fill the research gap in the nonlinear mechanism of TCM hospital payment reform; ③ Practice enlightenment: implement dynamic payment coefficient for hospitals with TCM service of >25% and give transitional subsidies to hospitals with TCM service of 15–25%; ④ Limitations and prospects: it is recognized that the sample size is small, and it can be expanded to 30+ tertiary TCM hospitals nationwide in the future. Combined with qualitative interviews, it will deepen the mechanism analysis.

## Data Availability

The original contributions presented in the study are included in the article/supplementary material, further inquiries can be directed to the corresponding author.
